# Upregulation of PGC-1α expression by pioglitazone mediates prevention of sepsis-induced acute lung injury

**DOI:** 10.1590/1414-431X2024e13235

**Published:** 2024-03-18

**Authors:** Jing Tang, Wenzhu Dong, Dan Wang, Qin Deng, Honggang Guo, Guibao Xiao

**Affiliations:** 1 Department of Infectious Diseases, Ziyang First People's Hospital, Ziyang, China; 2 Zhejiang Provincial Key Laboratory of Laboratory Animals and Safety Research, Hangzhou Medical College, Hangzhou, China

**Keywords:** Pioglitazone, Sepsis-induced acute lung injury, Peroxisome proliferator-activated receptor gamma coactivator 1-alpha, Macrophage polarization, Inflammation

## Abstract

The imbalance between pro-inflammatory M1 and anti-inflammatory M2 macrophages plays a critical role in the pathogenesis of sepsis-induced acute lung injury (ALI). Peroxisome proliferator-activated receptor gamma coactivator 1-alpha (PGC-1α) may modulate macrophage polarization toward the M2 phenotype by altering mitochondrial activity. This study aimed to investigate the role of the PGC-1α agonist pioglitazone (PGZ) in modulating sepsis-induced ALI. A mouse model of sepsis-induced ALI was established using cecal ligation and puncture (CLP). An *in vitro* model was created by stimulating MH-S cells with lipopolysaccharide (LPS). qRT-PCR was used to measure mRNA levels of M1 markers iNOS and MHC-II and M2 markers Arg1 and CD206 to evaluate macrophage polarization. Western blotting detected expression of peroxisome proliferator-activated receptor gamma (PPARγ) PGC-1α, and mitochondrial biogenesis proteins NRF1, NRF2, and mtTFA. To assess mitochondrial content and function, reactive oxygen species levels were detected by dihydroethidium staining, and mitochondrial DNA copy number was measured by qRT-PCR. In the CLP-induced ALI mouse model, lung tissues exhibited reduced PGC-1α expression. PGZ treatment rescued PGC-1α expression and alleviated lung injury, as evidenced by decreased lung wet-to-dry weight ratio, pro-inflammatory cytokine secretion (tumor necrosis factor-α, interleukin-1β, interleukin-6), and enhanced M2 macrophage polarization. Mechanistic investigations revealed that PGZ activated the PPARγ/PGC-1α/mitochondrial protection pathway to prevent sepsis-induced ALI by inhibiting M1 macrophage polarization. These results may provide new insights and evidence for developing PGZ as a potential ALI therapy.

## Introduction

Sepsis is a medical condition characterized by life-threatening organ dysfunction resulting from an abnormal and uncontrolled response of the body to an infection, and represents a major global public health problem (1). The lungs are particularly vulnerable to sepsis, and ensuing pulmonary dysfunction can culminate in acute lung injury (ALI) or even acute respiratory distress syndrome (1-4). Despite rapid advances in antimicrobial therapy and organ support, mortality from sepsis-induced ALI remains high. This may relate to dysregulated immune responses and imbalanced inflammation in sepsis-induced ALI.

As a key component of the innate immune system, macrophages play a pivotal role in inflammatory responses. It is increasingly recognized that macrophages are heterogeneous, and their polarized states are implicated in the pathogenesis of ALI (5,6). For instance, pro-inflammatory M1 macrophages promote free radical and cytokine production, exacerbating ALI, whereas M2 activation facilitates inflammation resolution. Hence, elucidating mechanisms governing macrophage polarization and promoting M2 skewing may improve sepsis-induced ALI.

Peroxisome proliferator-activated receptor gamma coactivator 1-alpha (PGC-1α) is a crucial transcriptional coactivator regulating cellular energy metabolism (7,8). Previous studies show aberrantly decreased PGC-1α expression in sepsis-induced ALI, suggesting its involvement in disease pathogenesis (9,10). Shifts in macrophage metabolism can influence polarization, and PGC-1α can enhance mitochondrial function to promote M2 macrophage polarization (11). This implies that PGC-1α may modulate macrophage M2 skewing by altering mitochondrial activity.

Pioglitazone (PGZ) is a drug in the thiazolidinedione class that is FDA-approved to treat type 2 diabetes (12). PGZ has been shown to up-regulate the expression of PGC-1α through peroxisome proliferator-activated receptor gamma (PPARγ) activation (13,14). This reveals a potential mechanism whereby PGZ may provide benefits beyond diabetes treatment, as PGC-1α activation enhances mitochondrial function and promotes M2 macrophage polarization (13,14). Some prior reports demonstrated PGZ could alleviate inflammatory responses in septic animal models (15-17). However, studies of PGZ's role in the PPARγ/PGC-1α/mitochondrial protection pathway during ALI progression remain limited. Here, we aimed to explore the effects and mechanisms of exogenous PGZ administration in sepsis-induced ALI using *in vivo* and *in vitro* approaches. This may provide new insights and evidence for developing PGZ as a potential therapy for sepsis-induced ALI.

## Material and Methods

### Antibodies, drugs, and reagents

Lipopolysaccharide (LPS) was obtained from Sigma Aldrich (China). PGZ was purchased from SelleckChem (China). The assays for the detection of tumor necrosis factor-α (TNF-α), interleukin (IL)-1β, IL-6, and IL-10 were purchased from Beyotime Biotech (China). The synthetic short interfering RNA (siRNA) targeting PGC-1α (siPGC-1α) and its corresponding negative control (NC) were purchased from GenePharma (China). The antibodies against PPARγ, nuclear respiratory factor 1 (NRF1), NRF2, mitochondrial transcription factor A (mtTFA), glyceraldehyde 3-phosphate dehydrogenase (GAPDH), and horseradish peroxidase (HRP)-conjugated goat anti-rabbit secondary antibody were purchased from ThermoFisher Scientific (USA).

### Establishment of ALI mice

In this study, we constructed an ALI mouse model (18) using cecal ligation and puncture (CLP) to explore whether PGZ preconditioning can attenuate ALI. This study was approved by the Animal Committee of Hangzhou Medical College (No. 2022-0117). The study utilized male C57BL/6 mice of SPF grade (Beijing Vital River Laboratory Animal Technology Co., Ltd., China), aged 6-8 weeks, with a body weight of 20±3 g. The mice were housed in the animal facility of Hangzhou Medical College, under a 12-h light/dark cycle and with *ad libitum* access to food and water. The mice were randomly divided into four groups: sham, PGZ, ALI, and ALI/PGZ.

To prepare a stock solution for the experiments, PGZ powder was dissolved in a saline solution containing 5% dimethyl sulfoxide (DMSO). The PGZ and ALI/PGZ groups received PGZ (18 mg/kg, which was suggested by previous research (19)) via intraperitoneal injection 1 h prior to sham (PGZ group) or CLP (ALI/PGZ group) surgery. In contrast, the sham and ALI groups were administered intraperitoneal injections of a saline solution containing 5% DMSO.

Prior to the CLP surgery, all mice were fasted for 12 h and water was withheld for 6 h. Each mouse was given an intraperitoneal injection of 2% sodium pentobarbital (50 mg/kg) in the lower right abdomen. Once satisfactory anesthesia was achieved, the mice were fixed in the supine position. The surgical site was prepared with routine skin preparation, sterilization, and draping. A vertical incision about 1 cm long was made 0.5 cm to the left of the midline of the abdomen. The peritoneum was bluntly separated to locate the cecum. The length of the cecum was measured with a ruler, and the middle portion of the cecum, located halfway from the blind end, was tied off. Avoiding the blood vessels, a 22-gauge needle was used to puncture the tied-off section twice, gently squeezing out a small amount of intestinal content. The cecum was then returned to the abdomen and the incision was sutured layer by layer. Postoperatively, 1 mL of warm (37°C) saline was injected subcutaneously in the nape of the neck for fluid resuscitation. The mice were then returned to separate cages in the animal room and allowed free access to food and water once they had recovered from anesthesia. The sham group underwent the same procedure, except for the cecal ligation and puncture. After surgery, the mice from each group were placed in cages with free access to food and water. At 24 h after surgery, one batch of mice (5 from each of the four groups) were euthanized, while the remaining mice (5 from each of the four groups) were continuously monitored for mortality for 7 days after surgery. The mice were euthanized using cervical dislocation, and the lung tissues were harvested immediately for subsequent experiments.

### Changes in wet/dry (W/D) proportion of lung

The mouse left lung surfaces were collected from the groups. The wet weight of the lung tissue was measured and recorded using an electronic scale. The weighed tissue was placed in an oven at 80°C for 48 h, until there were no more changes in weight. The dry weight of the lung tissue was then measured and recorded, and the W/D proportion was obtained.

### Cell culture and grouping

The murine alveolar macrophage MH-S cells were purchased from the Cell Bank of Chinese Academy of Sciences (China). Cells were cultured in Dulbecco's modified Eagle's medium supplemented with 10% fetal bovine serum (ThermoFisher), 100 IU/mL penicillin (Sigma Aldrich, USA), and 100 μg/mL streptomycin (Sigma Aldrich) under 37°C and 5% CO_2_.

MH-S cells (4×10^5^ cells/well) at the logarithmic phase of growth were inoculated in a 6-well cell culture plate. These cells were divided into five groups: blank (no treatment), LPS (10 μg/mL LPS), PGZ (10 μg/mL LPS + 10 μM PGZ), NC (10 μg/mL LPS + 10 μM PGZ + 200 nM NC), and siPGC-1α (10 μg/mL LPS + 10 μM PGZ + 200 nM siPGC-1α).

### Enzyme-linked immunosorbent assay (ELISA)

Mouse lung tissues (100 mg) collected from each group of mice were homogenized and centrifuged (10,000 *g*, 10 min, 4°C), and the supernatant was collected. The samples were then subjected to ELISA by adding reagents (TNF-α, IL-1β, and IL-6) according to the instructions of the assay kit, and the corresponding absorbance (450 nm) was measured using ultraviolet-visible spectroscopy (UV-1900i, Shimadzu, Japan).

### Hematoxylin and eosin (H&E) staining

After euthanizing the mice, the left lung tissue collected from each group of mice was extracted and fixed in 10% neutral buffered formalin solution at a volume ratio of 1:10 for preservation. Following embedding, 4-μm thick tissue sections were prepared. The sections were then stained with H&E and histopathological evaluation of myocardial tissue alterations under a BX61 microscope (Olympus, Japan) were carried out in three randomly selected fields by two pathologists in a blinded manner.

### Dihydroethidium (DHE) staining

Reactive oxygen species (ROS) levels in MH-S cells or in the lung tissues of mice were evaluated with the fluorescent dye DHE kit (Beyotime Biotech, China). MH-S cells in 24-well plates or the left lung tissue cyrosections collected from mice were incubated with 10 μM DHE at 37°C for 30 min. After being rinsed in phosphate-buffered saline three times, the fluorescent images were recorded using an Olympus DP70 digital camera (Japan) coupled to an Olympus IX71 inverted microscope (Japan). Fluorescence expression efficiency was measured using ImageJ 1.52p software (USA).

### Quantitative real-time polymerase chain reaction (qRT-PCR)

Total RNA was extracted from the lung tissues of each group of mice or from each group of cells using TRIzol reagent (ThermoFisher). The cDNA was synthesized from 1 μg of total RNA using the TaKaRa RNA PCR Kit (Takara, Japan). The qRT-PCR was performed with the TaqMan fluorogenic PCR system (Takara). After amplification, the Ct values of each group were recorded, and the relative gene expression was evaluated using the 2ˆ^-ΔΔCt^ method. The primer sequences were synthesized by Sangon (China) and are listed in Table 1.

**Table 1:** Primers used for qRT-PCR.

Gene symbol	Forward primer (5'→3')	Reverse primer (5'→3')
18s rRNA	TGTGCCCTGCTGAGCTCTCT	CAGACCCTCATCTCGTTTCCT
PGC-1α	CGGTGGATGAAGACGGATTGCC	ATTGTAGCTGAGCTGAGTGTTGGC
iNOS	CCTTCCGAAGTTTCTGGCAGCAGCG	GGCTGTCAGAGCCTCGTGGCTTTGG
MHC-II	GCCTGAAGCAGCAGATGAATG	CACACTGGGGCTTGTAGGAA
ArgI	GTGAAGAACCCACGGTCTGT	GCCAGAGATGCTTCCAACTG
CD206	GAAGCCAAGGTCCAGAAA	TGTTGAAAGCGTATGTCCA

### Detection of the copy number of mitochondrial DNA (mtDNA)

Total DNA of MH-S cells or the left lung tissue collected from mice was extracted using the DNeasy tissue kit (Qiagen, China). The copy number of mtDNA was detected by using qRT-PCR. For mtDNA, the upstream primer was 5′-ATCCTCCCAGGATTTGGAAT-3′, and the downstream primer was 5′-ACCGGTAGGAATTGCGATAA-3′. For genomic DNA (gDNA), the upstream primer was 5′-GGGAAGTCTTA-GGGAGGAGCA-3′, and the downstream primer was 5′-AGCTCTCAA-GAACTGTGCCC-3′. mtDNA/gDNA ratio was determined by dividing the relative quantity of mtDNA by the relative quantity of gDNA.

### Western blot analysis

The lung tissues collected from each group of mice and each group of cells were lysed with NE-PER™ nuclear and cytoplasmic extraction reagents (ThermoFisher) and complete protease inhibitor (Roche, Switzerland) on ice. The protein concentrations were determined using the Bicinchoninic Acid Protein Assay Kit (Keygen, China). For western blot analysis, 30 μg of protein from each sample was applied for sodium dodecyl-sulfate polyacrylamide gel electrophoresis. After separation, the proteins were transferred into polyvinylidene difluoride (PVDF) membranes (Roche, China). The PVDF membranes were blocked with 5% nonfat dry milk for almost 1 h and then incubated with the primary antibodies at 4°C for about 24 h. The following primary antibodies were used: anti-NRF1 (1:300), anti-NRF2 (1:300), anti-mtTFA (1:500), anti-p-PPARγ (1:200), anti-PPARγ (1:500), and anti-GAPDH (1:10000). This was followed by the incubation with the HRP-conjugated goat anti-rabbit secondary antibody (1:5000) at room temperature for about 1 h. Protein bands were visualized using BeyoECL Moon (Keygen). The band densities of western blot analysis were quantified by the ImageJ software (NIH, USA), and the relative protein levels were calculated based on GAPDH as the loading control.

### Statistical analysis

Statistical analysis was performed using GraphPad Prism 8.0 software (GraphPad Software, USA). Data normality was determined by using Shapiro-Wilk test. Statistical comparisons were made by one-way analysis of variance (ANOVA) with Tukey's range test. An adjusted P<0.05 was considered significant after Tukey's corrections for multiple comparisons.

## Results

### Effects of PGZ on survival and lung histopathological changes in ALI mice

**Figure 1:**
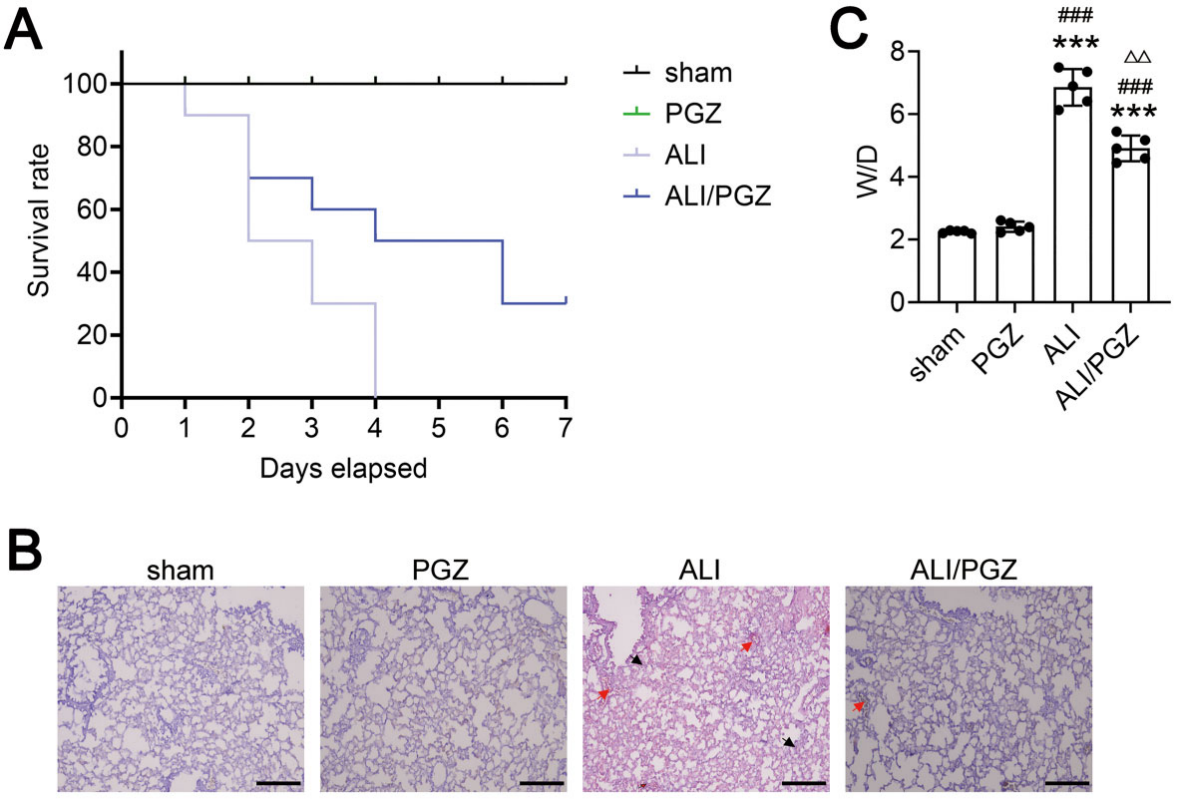
Pioglitazone (PGZ) treatment alleviates sepsis-induced acute lung injury (ALI) in mice. **A**, Analysis of the 7-day survival rate in the sham group, PGZ group, ALI group, and ALI/PGZ group of mice. **B**, H&E staining was used to detect pathological changes in the lung tissues. Scale bar, 50 μm. Red arrows indicate interstitial thickening, pulmonary edema, and blood cell exudation, and black arrows indicate inflammatory cells infiltration. **C**, Wet/dry (W/D) weight ratio in lung tissues. A total of five mice were included in each experimental group. The data are reported as means±SD of five independent experiments. ANOVA was performed followed by Tukey's range test for *post hoc* comparisons between groups. ***P<0.001 ALI and ALI/PGZ groups *vs* sham group. ^###^P<0.001 ALI and ALI/PGZ groups *vs* PGZ group. ^ΔΔ^P<0.01 ALI/PGZ group *vs* ALI group.

At 6 h post-surgery, all mice displayed symptoms of lethargy, piloerection, and diarrhea. Survival was 100% in the sham and PGZ groups at 7 days post-surgery, while survival was significantly decreased in the ALI and ALI/PGZ groups (Figure 1A, P<0.001). Specifically, survival rate at day 2 post-surgery was 50% in the ALI group and 70% in the ALI/PGZ group. By day 4, survival was 0% in the ALI group and 50% in the ALI/PGZ group, and by day 7, 30% in the ALI/PGZ group (Figure 1A).

H&E staining (Figure 1B) showed normal lung structure and intact, clear alveoli without exudate or inflammatory cell infiltration in the sham and PGZ groups. In contrast, the ALI group exhibited extensive inflammatory cell infiltration in the lung interstitium and alveolar spaces, thickened alveolar walls, fluid exudation in alveoli, ruptured septa, and fused or collapsed alveoli. The ALI/PGZ group of mice exhibited much less inflammatory cell infiltration in lung tissues than ALI mice, as well as a significant improvement in alveolar septum and pulmonary interstitial emphysema. Lung W/D weight ratio analysis also showed significantly increased ratios in the ALI and ALI/PGZ groups compared to the sham or PGZ groups. Moreover, the W/D ratio was markedly lower in the ALI/PGZ *vs* ALI group (Figure 1C).

### Effects of PGZ on the expression of PGC-1α and inflammatory cytokines in ALI mice

**Figure 2:**
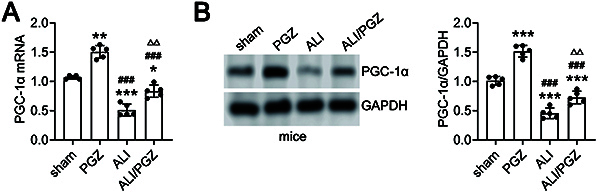
Pioglitazone (PGZ) treatment upregulates peroxisome proliferator-activated receptor gamma coactivator 1-alpha (PGC-1α) expression in the lung tissues. **A** and **B**, The expression levels of PGC-1α were detected by qRT-PCR (**A**) and western blot (**B**) in the lung tissues of the sham group, PGZ group, acute lung injury (ALI) group, and ALI/PGZ group of mice. A total of five mice were included in each experimental group. The data are reported as means±SD of five independent experiments. ANOVA was performed followed by Tukey's range test for *post hoc* comparisons between groups. *P<0.05, **P<0.01, ***P<0.001 PGZ, ALI, or ALI/PGZ groups *vs* sham group. ^###^P<0.001 ALI or ALI/PGZ groups *vs* PGZ group. ^ΔΔ^P<0.01 ALI/PGZ group *vs* ALI group.

The qRT-PCR analysis (Figure 2A) showed that compared to the sham group, the mRNA expression of PGC-1α in lung tissues was significantly increased in the PGZ and ALI/PGZ groups, but markedly decreased in the ALI group. Compared to the ALI group, PGC-1α mRNA expression was markedly enhanced in the ALI/PGZ group. Similar results were obtained by western blot (Figure 2B), which showed that PGC-1α protein levels were significantly higher in the PGZ and ALI/PGZ groups *vs* the sham group, but significantly lower in the ALI group. Compared to the ALI group, PGC-1α protein levels were significantly increased in the ALI/PGZ group (Figure 2B).

**Figure 3:**
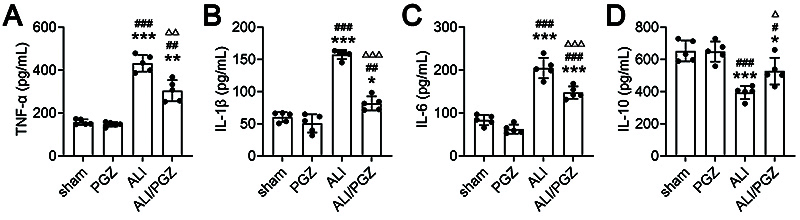
Pioglitazone (PGZ) treatment reduces the inflammatory factors expression in the lung tissues of acute lung injury (ALI) mice. The levels of tumor necrosis factor (TNF)-α (**A**), interleukin (IL)-1β (**B**), IL-6 (**C**), and IL-10 (**D**) were detected by ELISA in in the lung tissues of the sham group, PGZ group, ALI group, and ALI/PGZ group of mice. A total of five mice were included in each experimental group. The data are reported as means±SD of five independent experiments. ANOVA was performed followed by Tukey's range test for *post hoc* comparisons between groups. *P<0.05 **P<0.01, ***P<0.001 ALI or ALI/PGZ groups *vs* sham group. ^##^P<0.01, ^###^P<0.001 ALI or ALI/PGZ groups *vs* PGZ group. ^Δ^P<0.01, ^ΔΔ^P<0.01, ^ΔΔΔ^P<0.001 ALI/PGZ group *vs* ALI group.

ELISA results (Figure 3A-D) showed that compared to the sham and PGZ groups, pro-inflammatory cytokines TNF-α, IL-1β, and IL-6 were markedly elevated in the ALI and ALI/PGZ groups, while anti-inflammatory IL-10 was significantly decreased. There were no significant differences in cytokine levels between the sham and PGZ groups. Compared to the ALI group, TNF-α, IL-1β, and IL-6 levels were markedly lower and IL-10 was significantly higher in the ALI/PGZ group (Figure 3A-D).

### Effects of PGZ on macrophage polarization in LPS-stimulated MH-S cells *in vitro*

**Figure 4:**
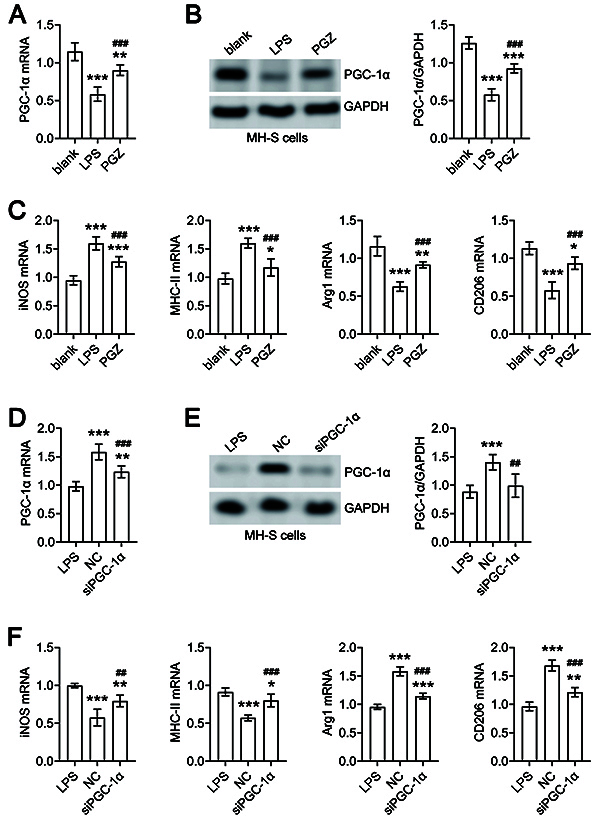
Pioglitazone (PGZ) treatment upregulates peroxisome proliferator-activated receptor gamma coactivator 1-alpha (PGC-1α) to prevent lipopolysaccharide (LPS)-stimulated macrophage M1 polarization. The expression levels of PGC-1α were detected by qRT-PCR (**A**) and western blot (**B**) in the blank, LPS, and PGZ groups of MH-S cells. **C**, The expression levels of iNOS, MHC-II, Arg1, and CD206 were detected by qRT-PCR in the blank, LPS, and PGZ groups of MH-S cells. *P<0.05, **P<0.01, ***P<0.001 LPS or PGZ groups *vs* blank group. ^###^P<0.001 PGZ group *vs* LPS group. The expression levels of PGC-1α were detected by qRT-PCR (**D**) and western blot (**E**) in the LPS, negative control (NC), and siPGC-1α groups of MH-S cells. **F**, The expression levels of iNOS, MHC-II, Arg1, and CD206 were detected by qRT-PCR in the LPS, NC, and siPGC-1α groups of MH-S cells. The data are reported as means±SD of three independent experiments. ANOVA was performed followed by Tukey's range test for *post hoc* comparisons between groups. *P<0.05, **P<0.01, ***P<0.001 NC or siPGC-1α groups *vs* LPS group. ^##^P<0.01, ^###^P<0.001 siPGC-1α group *vs* NC group.

The qRT-PCR and western blot analysis showed that PGC-1α expression was significantly decreased in the LPS group compared to the blank (no treatment) group of MH-S cells, but markedly rescued in the PGZ (LPS+PGZ) group (Figure 4A and B). qRT-PCR results also demonstrated that compared to the blank group, the mRNA levels of the M1 macrophage markers inducible nitric oxide synthase (iNOS) and major histocompatibility complex class II (MHC-II) were markedly elevated, while the mRNA expression of the M2 macrophage markers arginase 1 (Arg1) and CD206 was significantly decreased in the LPS and PGZ groups of MH-S cells. Compared to the LPS group, iNOS and MHC-II mRNA levels were markedly reduced and Arg1 and CD206 were significantly increased in the PGZ group of MH-S cells (Figure 4C).

Next, after siRNA-mediated PGC-1α knockdown, we observed that PGC-1α mRNA and protein levels were significantly decreased in the siPGC-1α group (LPS+PGZ+siPGC-1α) of MH-S cells compared to the NC (LPS+PGZ+NC) group of MH-S cells (Figure 4D and E). qRT-PCR analysis of iNOS, MHC-II, Arg1, and CD206 demonstrated that compared the NC group, iNOS and MHC-II expressions were markedly increased, while Arg1 and CD206 were significantly decreased in the LPS and siPGC-1α group of MH-S cells (Figure 4F). The mRNA expression of iNOS, MHC-II, Arg1, and CD206 were similar between the LPS and siPGC-1α groups of MH-S cells (Figure 4F). These results indicated that PGZ prevented LPS-induced M1 macrophage polarization in MH-S cells by upregulating PGC-1α expression.

### PGZ treatment activated the PPARγ/PGC-1α/mitochondrial protection pathway in LPS-stimulated MH-S cells *in vitro* and in ALI mice

**Figure 5:**
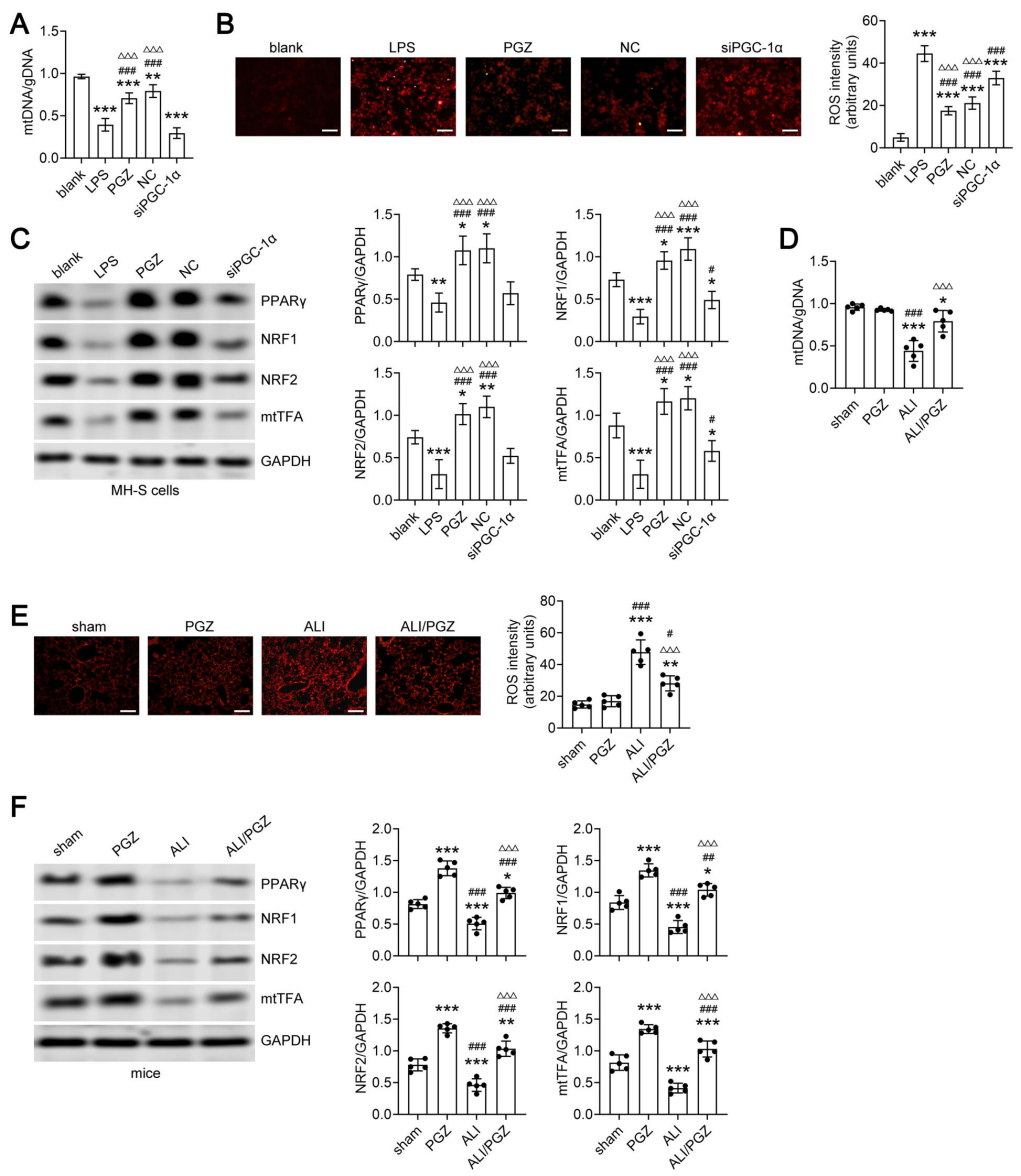
Pioglitazone (PGZ) treatment upregulated peroxisome proliferator-activated receptor gamma coactivator 1-alpha (PGC-1α) to modulate the expression of PPARγ and protect mitochondrial content and function. **A**, The quantitative analysis of mtDNA copy number were detected by qRT-PCR in the blank, lipopolysaccharide (LPS), PGZ, negative control (NC), and siPGC-1α groups of MH-S cells. **B**, The cellular reactive oxygen species (ROS) levels were detected by DHE staining in the blank, LPS, PGZ, NC, and siPGC-1α groups of MH-S cells. Scale bar, 50 μm. Right panel, the semi-quantification analysis of fluorescence intensity of DHE staining (three independent experiments). **C**, The expression levels of PPARγ and mitochondrial biogenesis-related proteins NRF1, NRF2, and mtTFA were detected by western blot in the blank, LPS, PGZ, NC, and siPGC-1α groups of MH-S cells. Right panel, graphs depict quantitative analysis of PPARγ, NRF1, NRF2, and mtTFA protein band densities (3 independent experiments). **D**, The relative mtDNA copy number was detected by qRT-PCR in the lung tissues of the sham group, PGZ group, acute lung injury (ALI) group, and ALI/PGZ group of mice. **E**, The cellular ROS levels were detected by DHE staining in the lung tissues of the sham group, PGZ group, ALI group, and ALI/PGZ group of mice. Scale bar, 50 μm. Right panel, the semi-quantification analysis of fluorescence intensity of DHE staining in the lung tissues. A total of five mice were included in each experimental group (five independent experiments). **F**, The expression levels of PPARγ and mitochondrial biogenesis-related proteins NRF1, NRF2, and mtTFA were detected by western blot in the lung tissues of the sham group, PGZ group, ALI group, and ALI/PGZ group of mice. Right panel, graphs depict quantitative analysis of protein band densities. A total of five mice were included in each experimental group (five independent experiments). ANOVA was performed followed by Tukey's range test for *post hoc* comparisons between groups. **A**-**C**, *P<0.05; **P<0.01; ***P<0.001 LPS, PGZ, NC, or siPGC-1α groups *vs* blank group. ^#^P<0.05; ^###^P<0.001 PGZ, NC, or siPGC-1α groups *vs* LPS group. ^ΔΔΔ^P<0.01 PGZ or NC groups *vs* siPGC-1α group. **D**-**F**, *P<0.05; **P<0.01; ***P<0.001 ALI or ALI/PGZ groups *vs* sham group. ^#^P<0.05; ^##^P<0.01; ^###^P<0.001 ALI or ALI/PGZ groups *vs* PGZ group. ^ΔΔΔ^P<0.01 ALI/PGZ group *vs* ALI group.

Previous research has demonstrated that PGZ can activate the expression of PGC-1α, most likely through the activation of PPARγ (13,14). Interestingly PGC-1α-mediated mitochondrial protection can further enhance the expression of PPARγ (20). In our study, we observed that LPS stimulation led to a reduction of mtDNA/gDNA ratio (mtDNA damage) in MH-S cells (Figure 5A). Furthermore, the levels of cellular ROS were elevated in LPS-stimulated MH-S cells (Figure 5B). Additionally, the expression of PPARγ and mitochondrial biogenesis-related proteins, including NRF1, NRF2, and mtTFA, was significantly reduced in the LPS group of MH-S cells compared to the blank group (Figure 5C). However, these alterations were noticeably restored in the PGZ (LPS+PGZ) and NC (LPS+PGZ+NC) groups of MH-S cells (Figure 5A-C). Notably, the expression of PPARγ, NRF1, NRF2, and mtTFA exhibited similar levels between the LPS group and the siPGC-1α group of MH-S cells (Figure 5C). Similar observations were assessed in ALI mice. We found that ALI mice showed mtDNA damage and ROS elevation in the lung tissue, whereas PGZ treatment significantly reduced mtDNA damage and ROS accumulation in the lung tissues of ALI mice (Figure 5D and E). Western blot analysis showed that the expression of PPARγ, NRF1, NRF2, and mtTFA were markedly increased in the PGZ and ALI/PGZ groups of mice compared to the sham group or the ALI group of mice (Figure 5F). These data revealed that PGZ may activate the PPARγ/PGC-1α/mitochondrial protection feedback loop to mitigate ALI.

## Discussion

Sepsis is a severe infectious complication, where immune dysfunction is implicated in pathogenesis. Growing evidence suggests that metabolic impairment in immune cells like macrophages, leading to immune dysfunction, are closely associated with sepsis (2-4). Mitochondria are the major sites of cellular energy metabolism, and mitochondrial dysfunction directly contributes to aberrant immune cell responses (21,22). PGC-1α is a crucial transcriptional coactivator of energy metabolism and mitochondrial oxidative phosphorylation (7,8). Downstream PGC-1α targets NRF1, NRF2, and mtTFA, which are key regulators of mitochondrial gene transcription and mtDNA replication, and their upregulation indicates enhanced mitochondrial biogenesis and function. Moreover, PGC-1α has been shown to play critical roles in regulating inflammation and M2 macrophage polarization (23,24). Exogenous expression of PGC-1α markedly enhanced macrophage recruitment and M2 skewing, whereas PGC-1α knockdown via siRNA abrogated this effect. In this study, we found that both PGC-1α expression and M2 macrophage polarization were aberrantly decreased in the lungs in sepsis mouse models, consistent with a previous report (9). These data suggested that PGC-1α may represent a therapeutic target for sepsis-induced injury.

PGZ is a drug used to improve insulin sensitivity in patients with type 2 diabetes (12). It works by activating PPARγ, a type of nuclear receptor that regulates the expression of genes involved in glucose and lipid metabolism. PGZ has the ability to activate PPARγ, thereby upregulating the expression of PGC-1α (13,14). This discovery elucidates a potential mechanism through which PGZ may confer advantages beyond its primary role in diabetes management. By activating PGC-1α, PGZ can enhance mitochondrial function and facilitate the polarization of M2 macrophages, implying a broader therapeutic use for PGZ (13,14). Previous studies have shown that PGZ could preserve mitochondrial function by inducing PGC-1α expression, thereby mitigating oxidative stress, injury, and inflammation in disease models (15-17,25,26). For example, intravenous PGZ in rats with cerebral ischemia substantially reduced infarct volume and improved neurological outcomes (25). Further *in vitro* experiments confirmed that PGZ protects neurons by augmenting PGC-1α expression and mitochondrial function, and upregulating antioxidant genes (25).

In this study, we found that intraperitoneal PGZ after sepsis induction significantly increased PGC-1α expression in lung tissues. Lung injury and edema were markedly improved in the sepsis-induced ALI mice treated with PGZ. Inflammatory cytokines like TNF-α, IL-1β, and IL-6 were decreased while the anti-inflammatory cytokine IL-10 was increased. *In vitro* experiments showed that PGZ upregulated M2 macrophage genes (*CD206* and *Arg1*) and downregulated M1 macrophage genes (*iNOS* and *MHC-II*), indicating it promoted M2 macrophage polarization. The effects of PGZ on macrophage polarization were abolished when PGC-1α was knocked down using siRNA. PGZ treatment also promoted the expression of NRF1, NRF2, and mtTFA in a PGC-1α-dependent manner. This suggested that PGZ may improve sepsis-induced ALI by inducing PGC-1α, which in turn inhibits inflammation and modulates macrophage polarization towards an anti-inflammatory phenotype through protecting mitochondrial function. Worth noting is that we observed that the upregulation of PPARγ expression during PGZ treatment also depended on PGC-1α, consistent with previous reports that PGC-1α accelerating mitochondrial biogenesis leads to elevated PPARγ expression (20). This suggested that PGZ treatment in sepsis establishes a PPARγ/PGC-1α positive feedback loop, which could further potentiate its effects of activating mitochondrial function and exerting anti-inflammatory actions.

In summary, our work found that PGZ treatment promoted PGC-1α expression and M2 macrophage polarization, likely via PPARγ/PGC-1α feedback loop-mediated mitochondrial protection. Thus, PGZ is a promising potential therapeutic agent for sepsis-induced ALI.
